# Efficacy and safety of remimazolam besylate in patients receiving mechanical ventilation: A randomized phase Ⅱa trial

**DOI:** 10.1016/j.jointm.2025.12.002

**Published:** 2026-01-08

**Authors:** Xiaobo Yang, Shouzhi Fu, Li Yu, Fengming Liang, Dezhong Li, Renhua Sun, Xiang Zhou, Xinting Yu, Luanyuan Tian, You Shang

**Affiliations:** 1 Department of Critical Care Medicine, Union Hospital, Tongji Medical College, Huazhong University of Science and Technology, Wuhan, Hubei, China; 2 Department of Critical Care Medicine, Wuhan Third Hospital, Wuhan, Hubei, China; 3 Department of Critical Care Medicine, The Central Hospital of Wuhan, Wuhan, Hubei, China; 4 Department of Critical Care Medicine, The Affiliated Wuxi People’s Hospital of Nanjing Medical University, Wuxi, Jiangsu, China; 5 Department of Critical Care Medicine, The Central Hospital of Enshi Tujia And Miao Autonomous Prefecture, Enshi, Hubei, China; 6 Department of Critical Care Medicine, Peolple's Hospital of Zhejiang, Hangzhou, Zhejiang, China; 7 Yichang Humanwell Pharmaceutical Co., Ltd, Yichang, Hubei, China

**Keywords:** Efficacy, Safety, Remimazolam besylate, Mechanical ventilation, Richmond Agitation-Sedation Scale, Adverse drug reaction

## Abstract

**Background:**

To preliminarily evaluate the efficacy, safety and continuous infusion dose of remimazolam besylate in short-term mechanically ventilated patients in intensive care units.

**Methods:**

This randomized phase Ⅱa trial was conducted in intensive care units (ICUs) in six hospitals in China. Adults that were anticipated to receive mechanical ventilation for ≥6 h and to have a Richmond Agitation–Sedation Scale (RASS) scores of -2 to +1 were enrolled in the trial and randomized (1:1:1) into three groups to receive 0.1, 0.2, or 0.4 mg/(kg·h) remimazolam besylate. The primary outcome was the percentage of time with the target RASS scores without rescue sedation. The secondary outcome was adverse drug reactions (ADRs). The optimal continuous infusion dose was also explored.

**Results:**

The percentage of time with the target RASS scores without rescue sedation did not differ among the groups (97.5±6.0 in the 0.1 mg/(kg·h) group with 11 participants, 95.2±8.0 in the 0.2 mg/(kg·h) group with 12 participants, and 97.0±2.4 in the 0.4 mg/(kg·h) group with 11 participants; *P*=0.665). The numbers of patients with ADRs were 7 (63.6%), 4 (33.3%), and 6 (54.5%) in the 0.1, 0.2, and 0.4 mg/(kg·h) groups, respectively (*P*=0.326). No participants experienced severe ADRs, and only 1 (9.1%) in the 0.4 mg/(kg·h) group experienced a grade 3–5 ADR. The optimal continuous infusion dose was 0.2–0.3 mg/(kg·h).

**Conclusions:**

Remimazolam besylate seems efficacious and safe in patients receiving short-term mechanical ventilation and light sedation, which justifies further trials.

**Trial Registration**Clinicaltrials.gov Identifier: NCT06124404.

## Introduction

Sedation is a fundamental treatment for most patients receiving invasive mechanical ventilation in the intensive care unit (ICU).^[^^1^^]^ Propofol and midazolam are the most commonly used sedatives in these patients. However, propofol is associated with the risk of bacterial contamination, injection-site pain, and delirium, and midazolam is associated with the risk of prolonged mechanical ventilation and delirium.^[^^1^^,^^2^^]^

Remimazolam is a new benzodiazepine being used for sedation in the ICU.^[^[3], [4]^]^ Similar to other benzodiazepines, it functions by directly agonizing gamma-aminobutyric acid (GABA) in the brain. Unlike other benzodiazepines, such as midazolam, which is metabolized by cytochrome P450-3A4, remimazolam is metabolized by nonspecific tissue esterase enzymes, making its metabolism organ independent.^[^^4^^,^^5^^]^ Carboxylesterase enzymes are the most important enzymes involved in the metabolism of remimazolam; these enzymes convert remimazolam to its major inactive metabolite (CNS7054).^[^^5^^]^ The onset of action of midazolam is 3–5 min, and the recovery time is approximately 2 h; however, recovery time is variable among different patients, especially patients with impaired renal function. The onset time and recovery time of patients who received remimazolam were much shorter than those who received midazolam.^[^[3], [4], [5]^]^ Burbery et al.^[^^6^^]^ coadministered remimazolam, midazolam and diazepam intravenously to six adult sheep and reported that remimazolam was undetectable in the plasma by 3 h but that midazolam and diazepam were detectable 10 h after injection.

However, the limited studies on remimazolam besylate are more focused on shorter durations during operations than on prolonged use in critically ill patients receiving mechanical ventilation in ICUs, and its optimal dose for sedation in ICUs is not well established. Previous data have been derived mainly from the induction and maintenance of general anesthesia and procedural sedation, whose predictive performance is poor in patients in ICUs.^[^^7^^,^^8^^]^ There are also two phase I studies on several-hour infusions in healthy participants.^[^^9^^,^^10^^]^ Earlier, we conducted a single-center study of continuous infusion of remimazolam besylate in noncardiac postoperative patients, but doses above 0.225 mg/(kg·h) were not evaluated.^[^^11^^]^ We aimed to preliminarily explore the efficacy, safety and potential optimal dose of remimazolam besylate in mechanically ventilated patients receiving relatively prolonged continuous infusion in ICUs rather than during operations.

## Methods

The study protocol was approved by the institutional review board of the leading center (Union Hospital, Tongji Medical College, Huazhong University of Science and Technology: 2021-1069-01) and each participating center. Written informed consent was obtained from all patients’ legal surrogates at inclusion and from all patients later when they were conscious and able to write, which allowed all patients to be blinded to the assignment until after the completion of the study intervention and weaned off the ventilator.

### Study design and study patients

This randomized phase Ⅱa trial (www.clinicaltrials.govNCT06124404) was conducted in intensive care units (ICUs) in six hospitals in China. Eligible participants were adults males and females aged between 18 and 80 years with a body mass index (BMI) between 18 and 30 kg/m^2^, who were intubated within 48 h and expected to require mechanical ventilation for ≥6 h and whose target Richmond Agitation-Sedation Scale (RASS) scores were between −2 and +1.

The following patients were excluded if they had any of the following conditions: allergy or unsuitability to any composition of study drugs or remifentanil; a living expectancy of less than 48 h; myasthenia gravis; status asthmaticus; abdominal compartment syndrome; serious hepatic dysfunction; chronic kidney disease with a glomerular filtration rate (GFR) <29 mL/(min·1.73m^2^); a possible requirement for tracheostomy within 24 h; a possible requirement for renal replacement therapy within 24 h; acute severe neurological disorder; any other condition interfering with sedation assessment; abuse of controlled substances or alcohol; pregnancy or lactation; and inclusion in another interventional trial in the past 30 days; or any other conditions deemed unsuitable for inclusion.

### Randomization and intervention

Randomization was performed using a centralized interactive web response system (IWRS) with six permuted blocks. The allocation sequence was concealed within the IWRS. Upon enrolling an eligible patient, the investigator accessed the IWRS, which irreversibly allocated the patient to one of the three treatment groups (0.1, 0.2, or 0.4 mg/(kg·h) remimazolam besylate in a 1:1:1 ratio).

Immediately after allocation, all preexisting analgesics and sedatives were stopped. A continuous remifentanil (Ruijie, Yichang Humanwell Pharmaceutical Co. Ltd.) infusion of 1.5 µg/(kg·h) was started. When the baseline RASS score reached ≥ +1, the participants received a loading dose of 0.08 mg/h remimazolam besylate (Ruima, Yichang Humanwell Pharmaceutical Co. Ltd.) given in 30 s, followed by a continuous infusion of 0.1 mg/(kg·h), 0.2 mg/(kg·h), or 0.4 mg/(kg·h) according to random allocation.

During the maintenance period, remimazolam besylate was titrated up if the RASS score was > +1 and down if the RASS score was <−2 at 0.1 mg/(kg·h). The minimum interval between the two adjustments was 5 min. When the dose of remimazolam besylate reached 1 mg/(kg·h), the dose of remifentanil was increased to 1.5 µg/(kg·h) if the RASS score was > +1. If the RASS score was <−2, the dose of remifentanil was decreased to 1.5 µg/(kg·h), and the dose of remimazolam besylate was decreased. If the maximum permitted doses of remimazolam besylate (2 mg/(kg·h)) and remifentanil (12 µg/(kg·h)) were reached and the RASS score remained > +1, additional sedatives could be initiated at the discretion of treating physicians. If necessary, the dose of remimazolam besylate could also be adjusted at the discretion of the treating physicians, and the RASS scores were evaluated before and after the adjustments.

Remimazolam besylate was discontinued if the patient was weaned off the ventilator or after 24 h of the study, whichever occurred first. All other care followed institutional standards.

### Data collection

The following items were collected from the participants: age, sex, height, body weight, Acute Physiology and Chronic Health Evaluation Ⅱ (APACHE Ⅱ) score, Sequential Organ Failure Assessment (SOFA) score, vital signs, and comorbidities.

RASS was assessed at baseline and, from the start of remimazolam besylate infusion, at 5 min, 10 min, 30 min, 1 h, and 2 h and every 2 h thereafter. Each dose adjustment triggered an additional RASS measurement, which was repeated every 5 min until the score was between −2 to +1. Upon discontinuation of remimazolam besylate, the RASS was recorded every 3 min until ≥ 0; the interval between stopping the infusion and the first RASS ≥ 0 was defined as the awakening time. The amounts of remifentanil and remimazolam besylate used during the study were recorded. The incubation time and any adverse drug reactions (ADRs) were also documented. Similar to the optimal infusion rate used by Doi et al.^[^^12^^]^, we also explored the optimal continuous infusion dose, which was defined as the dose with the longest duration during the maintenance period to achieve RASS scores between −2 and +1.

Blood samples were collected immediately before the first dose adjustment of remimazolam besylate, immediately before the first dose adjustment of remifentanil and whenever RASS scores were maintained between −2 and +1 for more than 30 min without any dose change. Blood samples were processed, and the concentrations of remimazolam besylate were measured according to methods validated in previous studies.^[^^9^^,^^13^^,^^14^^]^

### Outcomes

The primary outcome was efficacy, expressed as the percentage of time with RASS scores between −2 and +1 without rescue sedation. The secondary outcomes were the number and severity of ADRs. ADRs were defined as newly occurring events or worsening conditions from baseline that could not be explained by the natural disease course and needed treatment. ADRs were graded according to standard regulatory definitions and criteria.^[^^15^^]^

### Statistical analysis

As an exploratory trial, sample size estimation was not performed. In accordance with similar studies and the general rule of thumb for exploratory studies, at least ten patients were included in each group.^[^^16^^]^

Continuous variables are reported as the mean±standard deviation or median (interquartile range [IQR]), and categorical variables are reported as counts (percentages). For normally distributed continuous variables with equal variance, one-way ANOVA followed by the least significant difference (LSD) test was used to explore differences among the three groups and between two groups. For other continuous variables, the Kruskal‒Wallis test was used. For categorical variables, the chi-square test was used.

All analyses were performed on the modified intention-to-treat population (i.e., all randomized patients who received any study drug). A two-tailed *P* value <0.05 was considered to indicate statistical significance. Analyses were conducted with SAS version 9.4 (SAS Institute, Cary, NC, USA) and Stata version 14 (StataCorp, College Station, TX, USA).

## Results

### Baseline characteristics

Thirty-four patients were enrolled and randomized to receive remimazolam besylate at 0.1 mg/(kg·h) (*n*=11), 0.2 mg/(kg·h) (*n*=12), or 0.4 mg/(kg·h) (*n*=11). They had a mean age of (58.9±13.4) years, 73.5% were male, and their mean body mass index was (23.4±2.4) kg/m^2^ (Table 1). All the patients were Chinese, and 97.1% were of Han ethnicity. Among these patients, the most common comorbidities were hypertension (44.1%), anemia (44.1%), carcinoma (35.3%) and coronary atherosclerosis (32.4%). The baseline SOFA score was 2.5 (IQR: 2.0–4.0), and the APACHE Ⅱ score was 10.0 (IQR: 6.0–13.0). The baseline vital signs and laboratory test results are presented in Supplementary Tables S1 and S2.

**Table 1:** Baseline and demographic characteristics of the included patients.

Characteristics	0.1 mg/(kg·h) group (*n*=11)	0.2 mg/(kg·h) group (*n*=12)	0.4 mg/(kg·h) group (*n*=11)	Overall (*n*=34)
Age (years)	58.0±18.5	60.8±11.6	57.8±10.0	58.9±13.4
Sex				
Male	11 (100.0)	7 (58.3)	7 (63.6)	25 (73.5)
Female	0	5 (41.7)	4 (36.4)	9 (26.5)
Body mass index (kg/m^2^)	22.8±2.4	23.2±2.6	24.3±2.3	23.4±2.4
Han ethnicity	11 (100.0)	11 (91.7)	11 (100.0)	33 (97.1)
Comorbidity				
Diabetes	1 (9.1)	2 (16.7)	1 (9.1)	4 (11.8)
Hypertension	6 (54.5)	6 (50.0)	3 (27.3)	15 (44.1)
Coronary atherosclerosis	1 (9.1)	6 (50.0)	4 (36.4)	11 (32.4)
Atrial fibrillation	1 (9.1)	1 (8.3)	1 (9.1)	3 (8.8)
Anemia	3 (27.3)	4 (33.3)	8 (72.7)	15 (44.1)
Carcinoma	2 (18.2)	5 (41.7)	5 (45.4)	12 (35.3)
APACHE Ⅱ score	10.0 (6.0–13.0)	11.0 (7.5–15.5)	10.0 (7.0–13.0)	10.0 (6.0–13.0)
SOFA score	2.0 (2.0–4.0)	3.5 (2.0–7.0)	2.0 (2.0–4.0)	2.5 (2.0–4.0)

Data are presented as *n*(%), median±standard deviation, or median (interquartile range).

APACHE Ⅱ: Acute physiology and chronic health evaluation Ⅱ; SOFA:Sequential organ failure assessment.

### The use of remimazolam besylate and remifentanil

**Figure 1:**
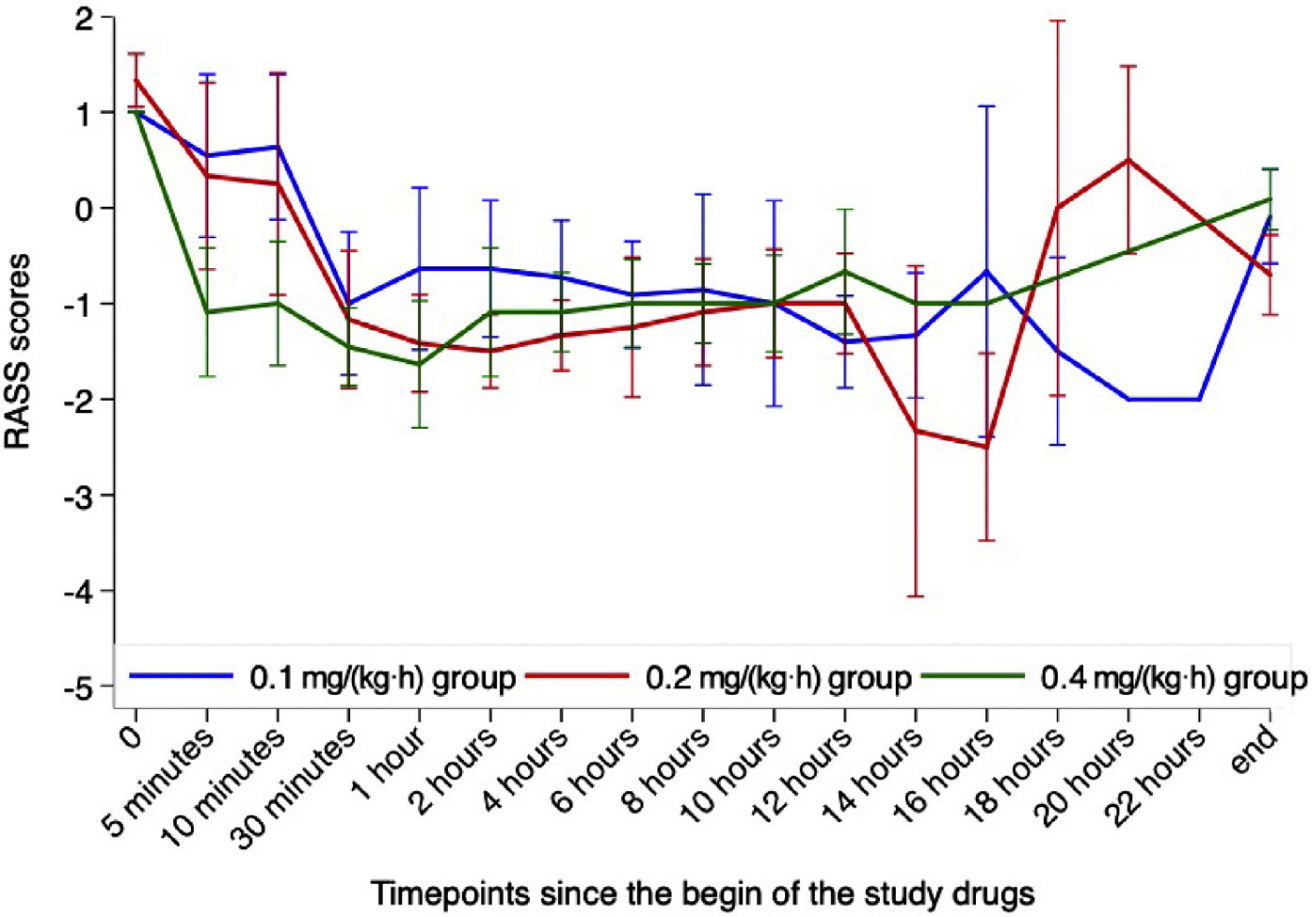
RASS scores among the three groups. The error bars represent 95% confidence intervals. RASS: Richmond agitation–sedation scale.

With the exception of four participants (33.3%) in the 0.2 mg/(kg·h) group, all the other participants had a RASS score of 1 immediately before the loading dose of remimazolam besylate. The RASS scores of the three groups at different timepoints are presented in Figure 1.

The amount of remifentanil was not significantly different among the three groups (*P*=0.410), nor was the duration of remimazolam besylate infusion (11.8 [IQR: 7.0–18.5] h in the 0.1 mg/(kg·h) group, 12.5 [IQR: 9.8–14.1] h in the 0.2 mg/(kg·h) group, or 10.5 [IQR: 9.2–12.4] h in the 0.4 mg/(kg·h) group; *P*=0.438) (Table 2). The total, loading, and maintenance doses of remimazolam besylate did not differ among the three groups. The number of patients who needed repeated loading doses was 4 (36.4%) in the 0.1 mg/(kg·h) group, 2 (16.7%) in the 0.2 mg/(kg·h) group, and 2 (18.2%) in the 0.4 mg/(kg·h) group (*P*=0.635). The times of remimazolam besylate titration did not differ among the three groups (2.0 [IQR: 0–13.0] in the 0.1 mg/(kg·h) group, 3.0 [IQR: 1.5–8.0] in the 0.2 mg/(kg·h) group and 5.0 [IQR: 1.0–9.0] group; *P*=0.898), and no patients received rescue sedation.

**Table 2:** The use of remimazolam besylate and remifentanil during the study.

Characteristics	0.1 mg/(kg·h) group (*n*=11)	0.2 mg/(kg·h) group (*n*=12)	0.4 mg/(kg·h) group (*n*=11)	*P* value
RASS score at the beginning of loading dose of remimazolam besylate				0.051
1	11 (100)	8 (66.7)	11 (100)	
2	0	4 (33.3)	0	
Amount of remifentanil (µg)	1393.947 (654.500–2186.800)	1694.700 (1253.575–3049.625)	1134.000 (1063.500–1766.000)	0.410
Duration of remimazolam besylate infusion (h)	11.8 (7.0–18.5)	12.5 (9.8–14.1)	10.5 (9.2–12.4)	0.438
Amount of remimazolam besylate				
Total (mg)	169.0 (50.2–382.8)	185.5 (110.1–396.3)	187.0 (131.4–276.1)	0.717
Loading dose (mg)	5.4 (4.6–6.2)	5.1 (4.4–5.6)	5.4 (4.8–5.6)	0.544
Maintaining dose (mg)	158.1 (45.0–377.6)	180.1 (110.1–396.3)	187.0 (131.4–276.1)	0.717
Number of patients needing boluses*	4 (36.4)	2 (16.7)	2 (18.2)	0.635
Number of boluses*	0 (0–1)	0 (0–0)	0 (0–0)	0.491
Times of titration of remimazolam besylate	2.0 (0–13.0)	3.0 (1.5–8.0)	5.0 (1.0–9.0)	0.898
Awakening interval (min)	1.0 (0–3.0)	2.0 (0–5.5)	0 (0–0)	0.116

Data are presented as *n*(%), or median (interquartile range).

RASS: Richmond agitation–sedation scale.

⁎ Boluses except for the loading dose.

The awakening interval was 1.0 (IQR: 0–3.0) min in the 0.1 mg/(kg·h) group, 2.0 (IQR: 0–5.5) min in the 0.2 mg/(kg·h) group, and 0 (IQR: 0–0) min in the 0.4 mg/(kg·h) group (*P*=0.116). All patients were successfully extubated.

The optimal continuous infusion dose was 0.20 (IQR: 0.10–0.30) mg/(kg·h) in the 0.1 mg/(kg·h) group, 0.25 (IQR: 0.20–0.40) mg/(kg·h) in the 0.2 mg/(kg·h) group, and 0.30 (IQR: 0.10–0.40) mg/(kg·h) in the 0.4 mg/(kg·h) group (*P*=0.410). The percentage of time with the optimal infusion dose was 84.1% (IQR: 53.7%–100%), 69.2% (IQR: 53.0%–93.9%), and 83.9% (IQR: 60.8%–97.0%) in the 0.1, 0.2, and 0.4 mg/(kg·h) groups, respectively (*P*=0.780). The concentrations of remimazolam besylate are presented in Supplementary Table S3.

### Primary outcome and secondary outcomes

The percentage of time with RASS scores between -2 and +1 without rescue sedation did not differ among the groups (97.5±6.0 in the 0.1 mg/(kg·h) group, 95.2±8.0 in the 0.2 mg/(kg·h) group, and 97.0±2.4 in the 0.4 mg/(kg·h) group; *P*=0.665)) (Table 3). The numbers of patients with ADRs were 7 (63.6%), 4 (33.3%), and 6 (54.5%) in the 0.1, 0.2 and 0.4 mg/(kg·h) groups, respectively (*P*=0.326). No participants experienced severe ADRs, and only 1 (9.1%) in the 0.4 mg/(kg·h) group experienced a grade 3–5 ADR, indicating a decrease in blood pressure. Different items of ADRs that occurred in more than 5% of all patients included are presented in Table 2. A decrease in blood pressure, an increase in blood pressure, an increase in bilirubin levels, a decrease in heart rate and an increase in heart rate occurred in 11 (32.4%), 4 (11.8%), 3 (8.8%), 2 (5.9%) and 2 (5.9%) participants, respectively, with no significant differences among the three groups.

**Table 3:** Primary and secondary outcomes.

Characteristics	0.1 mg/(kg·h) group (*n*=11)	0.2 mg/(kg·h) group (*n*=12)	0.4 mg/(kg·h) group (*n*=11)	*P* value
Primary outcome				
The percentage of time with RASS scores between −2 and +1 without rescue sedation	97.5±6.0	95.2±8.0	97.0±2.4	0.665
Secondary outcomes				
ADRs	7 (63.6)	4 (33.3)	6 (54.5)	0.326
Grade 3–5 ADRs	0	0	1 (9.1)	0.589
ADRs with treatment	1 (9.1)	1 (8.3)	4 (36.4)	0.141
ADRs with a decrease in remimazolam besylate levels	0	1 (8.3)	3 (27.3)	0.125
Items of ADRs				
Decrease in blood pressure	5 (45.5)	2 (16.7)	4 (36.4)	0.318
Increase in blood pressure	1 (9.1)	2 (16.7)	1 (9.1)	0.807
Increase in total bilirubin levels	1 (9.1)	0	2 (18.2)	0.307
Decrease in heart rate	0	1 (8.3)	1 (9.1)	0.600
Increase in heart rate	2 (18.2)	0	0	0.108

Data are presented as *n*(%), or mean±standard deviation.

ADRs: Adverse drug reactions; RASS: Richmond agitation–sedation scale.

## Discussion

In this trial of 34 postoperative patients who received mechanical ventilation, the percentage of time with RASS scores between −2 and +1 without rescue sedation was above 95%, and no severe ADRs occurred. A dosage of 0.20–0.30 mg/(kg·h) appears to be a good starting point for continuous infusion in further trials.

The safety of continuous remimazolam besylate infusion has also been compared with that of continuous propofol infusion in several studies. In 220 older hypertensive patients who underwent gastroenteroscopy, Gu et al.^[^^17^^]^ reported that compared with propofol, remimazolam conferred superior hemodynamic stability and resulted in faster recovery. In our two pilot studies, both remimazolam besylate and propofol were efficacious at achieving light to moderate sedation and deep sedation in mechanically ventilated patients in our ICU, without significant differences in the incidence of adverse events.^[^^18^^,^^19^^]^ In a single-center study with 80 older patients with a mean duration of mechanical ventilation for 4–5 days in the ICU, Li et al.^[^^20^^]^ reported no differences in in-hospital mortality or the incidence of adverse events between the two agents.

We observed no severe ADRs and only 1 grade 3–5 ADR occurred in the 0.4 mg/(kg·h) group. A decrease in blood pressure was the most common hemodynamic disturbance, occurring in approximately 1/3 of participants, which indicates that close monitoring of blood pressure is necessary during clinical application, particularly when higher doses are used. Similar safety profiles have been reported previously. In a phase I study with 9 Chinese volunteers receiving a continuous infusion of remimazolam besylate, no severe adverse events were noted, and only two episodes of hypoxemia responded promptly to supplemental oxygen.^[^^9^^]^ In a phase I study in German males, a total of 28 short episodes of SpO_2_ less than 93% occurred in 15 subjects, all of which were successfully treated by oxygen administration via a nasal cannula or by manual elevation of the lower jaw.^[^^10^^]^ Although the mean arterial blood pressure decreased 24%±6% from baseline, the lowest systolic arterial blood pressure was 96±8 mmHg during the continuous infusion of remimazolam.^[^^10^^]^ In 80 patients who underwent endoscopic retrograde cholangiopancreatography, Xiao et al.^[^^21^^]^ reported that compared with continuous infusion of propofol, remimazolam was associated with fewer cases of hypotension and smaller degrees of blood pressure decrease during approximately 40 min of procedures. In a study of surgical patients classified as American Society of Anesthesiologists Class Ⅲ and maintained on remimazolam at 1–2 mg/(kg·h) for general anesthesia, Doi et al.^[^^12^^]^ reported no severe ADRs, and blood pressure decreased in 21 out of 62 (33.9%) patients during surgery. In a phase Ⅱ, single-center, nonrandomized, open-label study, Grillot et al.^[^^22^^]^ infused remimazolam for ≤48 h in 30 critically ill patients and reported that plasma concentrations remained stable during infusion and declined rapidly after cessation; however, 50–84 adverse events occurred in 23 patients, including 11 serious events in eight patients, which were probably associated with patient severity (median simplified acute physiological score Ⅱ 38 [IQR: 30–46]) and the deep sedation target in 14 patients.

The dosing of remimazolam besylate in short-duration infusions has been explored in previous studies. In a phase I study in healthy Chinese volunteers, Sheng et al.^[^^9^^]^ reported that a loading dose of remimazolam besylate ≥0.075 mg/kg produced peak sedation within 1–2 min, and a 2-h continuous infusion induced deeper sedation and a more rapid recovery than midazolam did. Schüttler et al.^[^^10^^]^ reported a mean context-sensitive halftime of 6.8 min after a 4-h infusion and 12 min after an 8-h infusion in healthy German males. In this study, we titrated remimazolam besylate and identified the doses with the longest durations within the target RASS range (-2 to +1) across the three groups. The optimal continuous infusion dose of 0.1–0.3 mg/(kg·h) remimazolam besylate identified in this study supports earlier findings of 0.125–0.150 mg/(kg·h) in our phase I study^[^^11^^]^ and 0.18 (IQR: 0.15–0.22) mg/(kg·h) in our pilot study.^[^^18^^]^ We therefore recommend starting with 0.20 mg/(kg·h) remimazolam besylate for continuous infusion and titrating it up or down thereafter to achieve light sedation in mechanically ventilated patients.

The strictly monitored RASS scores made it possible for us to calculate the proportion of time within the target RASS range (−2 to +1) as the cumulative duration of qualifying intervals divided by the total infusion time for each participant. After every dose was titrated, the RASS was assessed every 5 min until the target was reached—a more frequent schedule than the fixed ≥2-h intervals recommended by the SCEPTER.^[^^23^^]^ We consider our approach more suitable for short-duration sedation studies but acknowledge that the short observation window is a limitation. Another limitation was the unblinded evaluation of the RASS. Although the RASS evaluators were not blinded, the absence of a control group means that any potential assessment bias was likely applied equally across all dose groups. The third limitation was the small sample size and the lack of a control group; however, both are not essential for a dose-selected phase Ⅱa study, and two larger randomized trials comparing remimazolam besylate with propofol are currently underway.^[^^24^^,^^25^^]^ The fourth limitation is the potential confounding effect of residual opioids administered during the surgery that preceded study enrollment. Although the protocol standardized analgesia during the trial, the variable types and doses of preoperative opioids could have influenced the baseline sedation requirements and the subsequent assessment.

## Conclusions

In this multicenter trial involving 34 postoperative mechanically ventilated adults, continuous remimazolam besylate infusion achieved the target sedation level (RASS scores −2 to +1 without rescue medication) for >95% of the study period across all three doses. No serious ADRs were observed. Remimazolam besylate appears to be efficacious and safe in patients receiving short-term mechanical ventilation and light sedation. The dose‒response data suggest that 0.20–0.30 mg/(kg·h) may represent an appropriate starting dose for further studies.

## Acknowledgments

None.

## Funding

This research did not receive any specific grant from funding agencies in the public, commercial, or not-for-profit sectors.

## Ethics Statement

The trial was approved by the ethics boards of all participating centers.

## CRediT authorship contribution statement

**Xiaobo Yang:** Writing – original draft, Investigation, Formal analysis, Data curation, Conceptualization. **Shouzhi Fu:** Validation, Investigation, Data curation. **Li Yu:** Validation, Investigation, Data curation. **Fengming Liang:** Validation, Investigation, Data curation. **Dezhong Li:** Validation, Investigation, Data curation. **Renhua Sun:** Validation, Investigation, Data curation. **Xiang Zhou:** Writing – review & editing, Supervision, Resources, Methodology, Formal analysis, Conceptualization. **Xinting Yu:** Writing – review & editing, Supervision, Resources, Project administration, Formal analysis, Conceptualization. **Luanyuan Tian:** Writing – review & editing, Validation, Supervision, Resources, Project administration, Methodology, Funding acquisition, Conceptualization. **You Shang:** Writing – review & editing, Validation, Supervision, Resources, Project administration, Funding acquisition, Formal analysis, Conceptualization.

## Conflict of Interest

This study was sponsored by Yichang Humanwell Pharmaceutical Co Ltd. It was conducted to generate preliminary data necessary for planning a pivotal phase Ⅲ clinical trial, which would ultimately support a supplement to the drug's package insert. Yichang Humanwell Pharmaceutical Co Ltd manufactures both remifentanil and remimazolam besylate. It provided both drugs and payments for SMO and statisticians. Xiang Zhou, Xinting Yu, and Luanyuan Tian work in Yichang Humanwell Pharmaceutical Co Ltd. Xiaobo Yang, Shouzhi Fu, Li Yu, and You Shang conduct other trials sponsored by Yichang Humanwell Pharmaceutical Co, Ltd. Xiaobo Yang and You Shang received drugs for free from Yichang Humanwell Pharmaceutical Co, Ltd for some invesigator-initiated trials.

Given his role as Editorial Board Member, You Shang had no involvement in the peer-review of this article and has no access to information regarding its peer-review. Full responsibility for the editorial process for this article was delegated to another journal editor.

## Data Availability

Data will be available 2 years after the publication of this manuscript upon a reasonable study protocol with an ethical permission.

## Appendix Supplementary materials

Image, application 1

## References

[1] Devlin J.W., Skrobik Y., Gélinas C., Needham D.M., Slooter A., Pandharipande P.P. (2018). Clinical practice guidelines for the prevention and management of pain, agitation/sedation, delirium, immobility, and sleep disruption in adult patients in the ICU. Crit Care Med.

[2] Ge Q.Y., Zheng C., Song X.B., Cong Z.Z., Luo J., Zheng H.T. (2025). The relationship between the average infusion rate of propofol and the incidence of delirium during invasive mechanical ventilation: a retrospective study based on the MIMIC IV database. CNS Neurosci Ther.

[3] Mathur R., Chittoria K., Sharma A., Goyal S., Kothari N. (2025). What every intensivist should know about Remimazolam. Indian J Crit Care Med.

[4] Hansen T.G., Engelhardt T. (2025). Remimazolam in children: a comprehensive narrative review. Anesthesiol Perioper Sci.

[5] Dessai S., Ninave S., Bele A. (2024). The rise of Remimazolam: A review of pharmacology, clinical efficacy, and safety profiles. Cureus.

[6] Burbery K., Brosnan R.J., Cenani A., Machado M., Knych H.K. (2025). Pharmacokinetics of remimazolam, midazolam and diazepam in sheep. Vet Anaesth Analg.

[7] Masui K., Stöhr T., Pesic M., Tonai T. (2022). A population pharmacokinetic model of remimazolam for general anesthesia and consideration of remimazolam dose in clinical practice. J Anesth.

[8] Eleveld D.J., Colin P.J., Van den Berg J.P., Koomen J.V., Stoehr T., Struys M. (2025). Development and analysis of a remimazolam pharmacokinetics and pharmacodynamics model with proposed dosing and concentrations for anaesthesia and sedation. Br J Anaesth.

[9] Sheng X.Y., Liang Y., Yang X.Y., Li L.E., Ye X., Zhao X. (2020). Safety, pharmacokinetic and pharmacodynamic properties of single ascending dose and continuous infusion of remimazolam besylate in healthy Chinese volunteers. Eur J Clin Pharmacol.

[10] Schüttler J., Eisenried A., Lerch M., Fechner J., Jeleazcov C., Ihmsen H. (2020). Pharmacokinetics and pharmacodynamics of Remimazolam (CNS 7056) after continuous infusion in healthy male volunteers: part I. Pharmacokinetics and clinical pharmacodynamics. Anesthesiology.

[11] Tang Y., Yang X., Shu H., Yu Y., Xu J., Pan S. (2022). Remimazolam besylate for sedation of postoperative patients in intensive care units: a phase I, open label, dose-finding study. Chin Med J (Engl).

[12] Doi M., Hirata N., Suzuki T., Morisaki H., Morimatsu H., Sakamoto A. (2020). Safety and efficacy of remimazolam in induction and maintenance of general anesthesia in high-risk surgical patients (ASA Class III): results of a multicenter, randomized, double-blind, parallel-group comparative trial. J Anesth.

[13] Wang X., Hu X., Bai N., Li L., Zhang M., Cheng Z. (2022). Safety and efficacy of remimazolam besylate in patients undergoing colonoscopy: A multicentre, single-blind, randomized, controlled, phase Ⅲ trial. Front Pharmacol.

[14] Zhou Y.Y., Yang S.T., Duan K.M., Bai Z.H., Feng Y.F., Guo Q.L. (2022). Efficacy and safety of remimazolam besylate in bronchoscopy for adults: A multicenter, randomized, double-blind, positive-controlled clinical study. Front Pharmacol.

[15] US Department of Health and Human Services. Common Terminology Criteria for Adverse Events (CTCAE) Version 5.0. Available from: https://dctd.cancer.gov/research/ctep-trials/for-sites/adverse-events/ctcae-v5-8x11.pdf. [Last accessed on 2025 November 8].

[16] Lancaster G.A., Dodd S., Williamson P.R. (2004). Design and analysis of pilot studies: recommendations for good practice. J Eval Clin Pract.

[17] Gu Q., Zeng D., Lin D., Zou J., Sun J., Wang H. (2025). Effect of Remimazolam versus Propofol on hemodynamics in elderly hypertensive patients undergoing gastroenteroscopy: A multicenter, randomized controlled clinical trial. Drug Des Devel Ther.

[18] Tang Y., Yang X., Yu Y., Shu H., Yuan Y., Liu H. (2022). Remimazolam besylate versus propofol for long-term sedation during invasive mechanical ventilation: a pilot study. Crit Care.

[19] Tang Y., Gao X., Xu J., Ren L., Qi H., Li R. (2023). Remimazolam besylate versus propofol for deep sedation in critically ill patients: a randomized pilot study. Crit Care.

[20] Li Y., Yuan Y., Zhou J., Ma L. (2025). Effect of remimazolam besylate on elderly patients with mechanical ventilation: a single-center randomized controlled study. BMC Anesthesiol.

[21] Xiao Y.Y., Zou H.D., Qin X.N., Zhu R., Dai R.P. (2025). A comparison of remimazolam versus propofol on blood pressure changes during therapeutic endoscopic retrograde cholangiopancreatography: A randomized controlled trial. Anesth Analg.

[22] Grillot N., Vourc'h M., Hourmant Y., Bouras M., Rozec B., Rouhani A. (2025). A phase 2 open-label pilot study of Remimazolam for sedation in critically ill patients. Anaesth Crit Care Pain Med.

[23] Ward D.S., Absalom A.R., Aitken L.M., Balas M.C., Brown D.L., Burry L. (2021). Design of clinical trials evaluating sedation in critically ill adults undergoing mechanical ventilation: recommendations from sedation Consortium on endpoints and procedures for treatment, education, and research (SCEPTER) recommendation III. Crit Care Med.

[24] Yang X., Tang Y., Du R., Yu Y., Xu J., Zhang J. (2023). Long-term sedation with remimazolam besylate versus propofol in critically ill patients during invasive mechanical ventilation: a study protocol for a multicenter randomized non-inferior trial. Front Pharmacol.

[25] Tang Y., Shu H., Ren L., Li R., Zou X., Qi H. (2025). Remimazolam besylate versus propofol for short-term sedation in critically ill patients receiving mechanical ventilation: protocol for a multicenter randomized non-inferior trial. Adv Ther.

