# Supplementary material for: Efficacy and safety of remimazolam besylate in patients receiving mechanical ventilation: A randomized phase Ⅱa trial
Source: J Intensive Med. 2026 Jan 8;6(3):277–83. doi: 10.1016/j.jointm.2025.12.002 (PMC13184453; doi:10.1016/j.jointm.2025.12.002)
Supplement: Supplementary file 1 [file mmc1.docx]

Table S1. The baseline vitals in the three groups

| Characteristics | 0.1 mg/(kg·h) group (n = 11) | 0.2 mg/(kg·h) group (n = 12) | 0.4 mg/(kg·h) group (n = 11) | pooled values （n = 34） |
| --- | --- | --- | --- | --- |
| Heart rate (beats per minute) | 92.5±15.44 | 72.1±16.81 | 88.6±22.11 | 84.0±19.93 |
| Systolic blood pressure (mmHg) | 125.2±19.51 | 121.8±23.28 | 118.7±17.25 | 121.9±19.83 |
| Diastolic blood pressure (mmHg) | 69.7±11.23 | 66.8±11.43 | 65.4±9.00 | 67.3±10.47 |
| Mean blood pressure (mmHg) | 83.1±12.05 | 84.4±14.22 | 80.3±8.92 | 82.6±11.77 |
| Respiratory rate (breathes per minute) | 15.5±2.54 | 15.9±3.26 | 16.1±3.65 | 15.9±3.10 |
| SpO_2_ (%) | 98.6±2.50 | 99.1±1.24 | 99.8±0.60 | 99.2±1.66 |

Data are presented as mean ± standard deviation.

Table S2. The baseline laboratory tests in the three groups

| Characteristics | 0.1 mg/(kg·h) group (n = 11) | 0.2 mg/(kg·h) group (n = 12) | 0.4 mg/(kg·h) group (n = 11) | pooled values  （n = 34） |
| --- | --- | --- | --- | --- |
| Blood routine |  |  |  |  |
| Hemoglobin (g/l) | 116.0±32.15 | 122.8±14.63 | 99.8±16.46 | 113.2±23.72 |

| Red blood cell (10^12^/l) | 3.858±1.045 | | | | 3.986±0.498 | | 3.485±0.674 | | | | 3.783±0.773 | | |
| --- | --- | --- | --- | --- | --- | --- | --- | --- | --- | --- | --- | --- | --- |
| Platelet (10^9^/l) | 186.1±58.91 | | | | 157.1±77.55 | | 221.5±100.92 | | | | 187.3±82.85 | | |
| White blood cell (10^9^/l) | 8.631±4.356 | | | | 11.118±3.583 | | 9.274±2.617 | | | | 9.716±3.644 | | |
| Neutrophil (10^9^/l) | 6.657±4.650 | | | | 9.794±3.573 | | 7.525±2.444 | | | | 8.045±3.803 | | |
| Lymphocyte (10^9^/l) | 1.325±0.738 | | | | 0.829±0.428 | | 1.143±0.587 | | | | 1.091±0.612 | | |
| Liver function |  | | | |  | |  | | | |  | | |
| AST (U/L) | 18.20 [15.0 -26.00] | | | | 41.50 [22.50 - 46.20] | | 30.50 [20.00 - 103.00] | | | | 24.00 [19.00 - 46.00] | | |
| ALT (U/L) | 15.00 [5.90 - 33.00] | | | | 34.00 [13.00 - 50.00] | | 21.00 [12.00 - 102.00] | | | | 21.50 [12.00 - 41.00] | | |
| Total bilirubin (μmol/L) | | 12.10 [5.60 - 24.50] | | | | 13.80 [10.85 - 16.65] | | 12.65 [8.10 - 15.60] | | | 13.70 [8.10 - 18.10] | | |
| Albumin (g/L) | 35.770 [30.500 - 41.600] | | | | 35.300 [31.950 - 38.950] | | 31.500 [23.900 - 33.430] | | | | 33.430 [30.500 - 38.400] | | |
| Potassium (mmol/L) | | 3.995±0.447 | | | | 3.964±0.541 | | 3.920±0.481 | | | 3.961±0.476 | | |
| Sodium (mmol/L) | 140.91±3.532 | | | | 141.28±3.830 | | 141.36±2.940 | | | | 141.18±3.362 | | |
| Chloride (mmol/L) | | 108.06±6.892 | | | | 109.55±1.978 | | 108.15±4.092 | | | | 108.60±4.684 | |
| Renal function |  | | | |  | |  | | | |  | | |
| Creatine (μmol/L) | | | 92.16±25.884 | | | 70.00±30.423 | | | 70.92±24.266 | | | 77.47±28.223 | |
| BUN (mmol/L) | | | | 7.542±6.543 | | 4.845±2.066 | | | | 5.115±1.399 | | | 5.805±4.060 |

Data are presented as mean ± standard deviation or median [interquartile range].

Table S3. The concentrations of remimazolam besylate in the three groups

| Characteristics | 0.1 mg/∙ group (n = 11) | 0.2 mg/(kg·h) group (n = 12) | 0.4 mg/(kg·h) group (n = 11) |
| --- | --- | --- | --- |
| Immediately before the first dose adjustment of remimazolam besylate | | | |
| Number | 6 | 9 | 10 |
| Concentration (ng/ml) | 177 [150 - 214] | 230.5 [180 - 462] | 266.5 [166 – 409] |
| Immediately before the first dose adjustment of remifentanil | | | |
| Number | 4 | 7 | 2 |
| Concentration (ng/ml) | 175.5 [99.5 – 260] | 469 [82.6 – 667] | 422 [206 – 638] |
| Whenever RASS was maintained between -2 to 1 for more than 30 minutes without any dose change | | | |
| Number | 10 | 12 | 11 |
| Concentration (ng/ml) | 135 [87.2 – 269] | 218 [146 – 362.5] | 201 [165 – 238] |

Data are presented as count or median [interquartile range].

Table S4. A full list of ADRs

| Items of ADRs | 0.1 mg/(kg·h) group (n = 11) | 0.2 mg/(kg·h) group (n = 12) | 0.4 mg/(kg·h) group (n = 11) | Pooled |
| --- | --- | --- | --- | --- |
| Decrease of blood pressure | 5(45.5) | 2(16.7) | 4(36.4) | 11(32.4) |
| Increase of blood pressure | 1(9.1) | 2(16.7) | 1(9.1) | 4(11.8) |
| Increase of total bilirubin | 1(9.1) | 0(0) | 2(18.2) | 3(8.8) |
| Decrease of heart rate | 0(0) | 1(8.3) | 1(9.1) | 2(5.9) |
| Increase of heart rate | 2(18.2) | 0(0) | 0(0) | 2(5.9) |
| Increase of white blood cell count | 0(0) | 0(0) | 1(9.1) | 1(2.9) |
| Prolongation of QT interval on ECG | 1(9.1) | 0(0) | 0(0) | 1(2.9) |
| Decrease of platelet count | 0(0) | 0(0) | 1(9.1) | 1(2.9) |
| Increase of neutrophil count | 0(0) | 0(0) | 1(9.1) | 1(2.9) |
| Hypokalemia | 0(0) | 1(8.3) | 0(0) | 1(2.9) |
| Urinary tract infection | 0(0) | 1(8.3) | 0(0) | 1(2.9) |
| Pyrexia | 0(0) | 0(0) | 1(9.1) | 1(2.9) |
| Ventricular premature complex | 0(0) | 0(0) | 1(9.1) | 1(2.9) |

Data are presented as count of patients (%). ADRs, adverse drug reactions; ECG, electrocardiogram.
